# Whole-body PET image denoising for reduced acquisition time

**DOI:** 10.3389/fmed.2024.1415058

**Published:** 2024-09-30

**Authors:** Ivan Kruzhilov, Stepan Kudin, Luka Vetoshkin, Elena Sokolova, Vladimir Kokh

**Affiliations:** ^1^Applied Mathematics and AI, Moscow Power Engineering Institute, Moscow, Russia; ^2^Sber AI Lab, Moscow, Russia; ^3^Moscow Institute of Physics and Technology, Dolgoprudny, Russia; ^4^LLC SberMedAI, Moscow, Russia

**Keywords:** artificial intelligence, positron emission tomography, SUV, noise reduction, MedNeXt, SwinIR

## Abstract

**Purpose:**

A reduced acquisition time positively impacts the patient's comfort and the PET scanner's throughput. AI methods may allow for reducing PET acquisition time without sacrificing image quality. The study aims to compare various neural networks to find the best models for PET denoising.

**Methods:**

Our experiments consider 212 studies (56,908 images) for 7MBq/kg injected activity and evaluate the models using 2D (RMSE, SSIM) and 3D (SUVpeak and SUVmax error for the regions of interest) metrics. We tested 2D and 2.5D ResNet, Unet, SwinIR, 3D MedNeXt, and 3D UX-Net. We have also compared supervised methods with the unsupervised CycleGAN approach.

**Results and conclusion:**

The best model for PET denoising is 3D MedNeXt. It improved SSIM on 38.2% and RMSE on 28.1% in 30-s PET denoising and on 16.9% and 11.4% in 60-s PET denoising when compared to the original 90-s PET reducing at the same time SUVmax discrepancy dispersion.

## 1 Introduction

Positron emission tomography (PET) is a molecular imaging technique that produces a three-dimensional radiotracer distribution map representing properties of biologic tissues, such as metabolic activity. Many patients undergo more than one PET/CT scan per year. According to the OECD/EU report (42), an average number of PET scans per 1,000 people is 3.3 in EU25 countries, with a maximum value of 10.2 in Denmark. The higher the injected activity, the less noise in the reconstructed images and the more radioactive exposure for a patient and for the healthcare operators.

The task of accelerating a PET/CT scan is to create an algorithm that takes a low-time PET image as input and converts it into an image with diagnostic quality that corresponding to a PET image with a standard exposure time. This task is equivalent to reducing the administered dose of a radio-pharmaceutical. Both of these tasks are noise reduction tasks (54).

**Figure 1:**
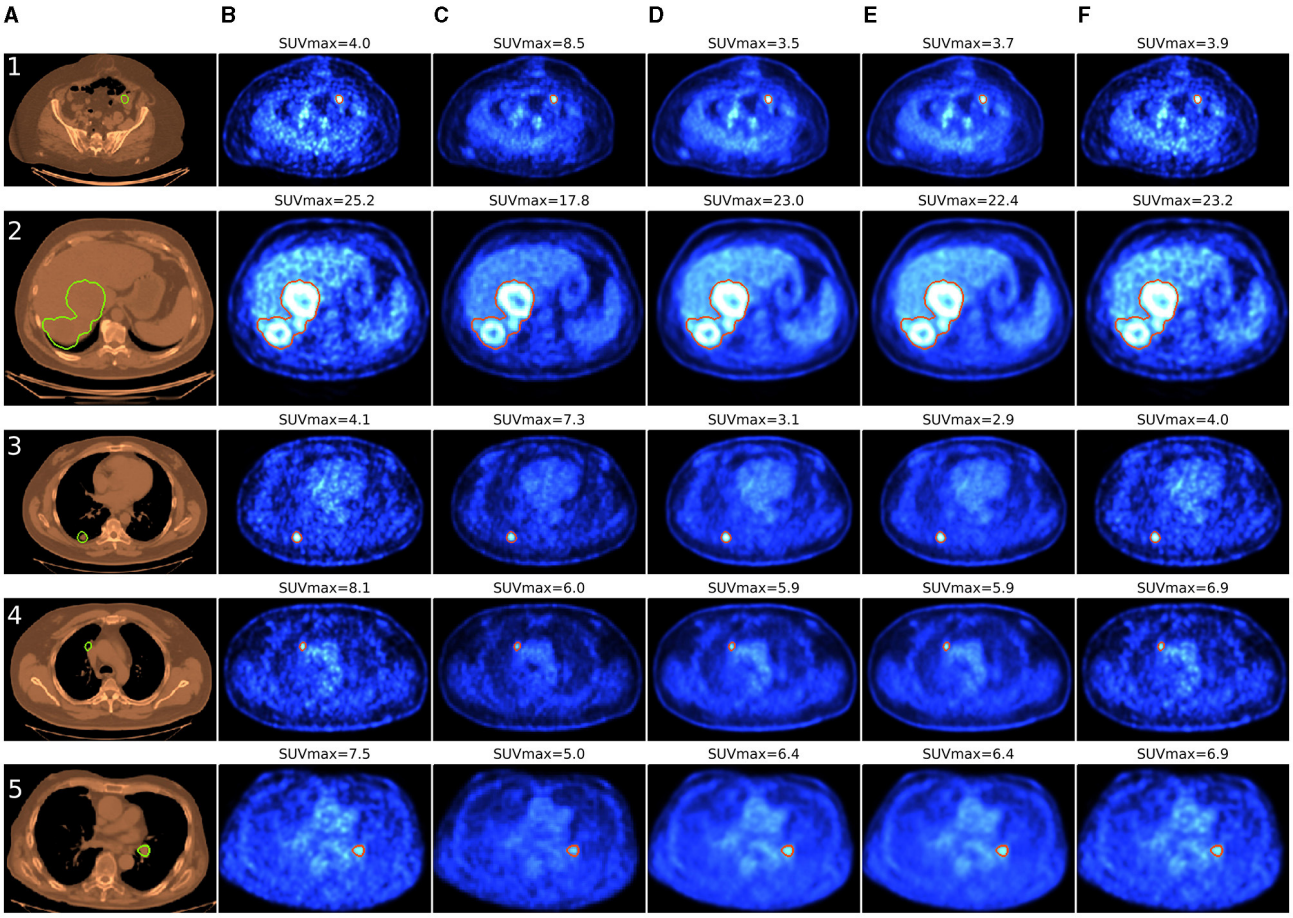
Comparison of denoising methods applied on low-time (30 s) PET reconstruction and SUVmax estimation within the region of interest. Tumor segmentation is done automatically by nnUnet using CT and PET. **(A)** CT **(B)** Low-time PET (30 s). **(C)** Full-time PET (90 s). **(D)** Reconstructed PET (by SwinIR 5 layers). **(E)** Reconstructed PET (by MedNeXt small). **(F)** Reconstructed PET (by Gaussian filter).

Deep learning methods may reduce injected activity or acquisition time by utilizing low-dose (LD)/low-time (LT) and full-dose (FD)/full-time (FT) images (Figure 1) to train models that can predict standard-dose images from LD/LT inputs. A reduced acquisition time positively impacts the patient's comfort or the scanner's throughput, which enables more patients to be scanned daily, lowering costs.

The drawback of the recent studies is the need for more comparison between a broad group of methods on a level playing field, especially between supervised and unsupervised methods. Moreover, as methods are tested on different data sets and for various PET time frames, comparing them is complicated. Furthermore, studies differ in the metrics used for denoising quality assessment, for example, some (20, 23, 59, 62) evaluate only image similarity metrics such as RMSE but do not take into account SUV characteristics.

Our study aims to overcome the drawbacks by finding the best backbone and model and comparing the performance of supervised and unsupervised methods for PET denoising. We tested supervised 2D and 2.5D methods [ResNet, Unet, SwinIR (31)] and 3D MedNeXt (49). We have also applied unsupervised pix2pix and CycleGAN with identity and image prior losses (36, 62). We reconstructed FT 90-s/bed position PET from LT PET 30 and 60 s/bed position. Our PET denoising is indicated for use in whole-body PET-CT in patients with primary staging and for assessing the dynamics of those with adenocarcinoma [with a proliferation index of more than 20% (ki67)] and melanomas.

### 1.1 Related works

The latest (as of 2024) review on the low-count PET reconstruction are the studies (8, 19). Early studies of PET denoising treated specific parts of the human body, such as the brain and lungs, and used small-size data sets to produce low-quality reconstructions. For example, Gong et al. (16) utilized pretrained VGG19 and perception loss for supervised denoising lungs and brains. One could find the comprehensive overview of methods before 2020 in (50). Table 1 represents the summary of the later studies on PET denoising. In the table, the sign * indicates the SUV metrics where the authors did not specify what kind of SUV—mean, peak, or max they estimated.

**Table 1:** Summarization of low-count PET denoising methods.

**References**	**Input data, number of studies**	**Network architecture**	**Low count, full count**	**Scanner model**	**Metrics**
Sanaat et al. (50)	Brain sinograms, 140	3D Unet	–, 20 min	Siemens biograph mCT	RMSE, SSIM, PSNR, SUV^*^ bias STD
Sanaat et al. (51)	Whole body, 100	Cycle GAN, ResNet	3 min, 27 min	Siemens biograph mCT	RMSE, SSIM, PSNR, Visual eval, SUV^*^ bias STD, R2
Sanaei et al. (52)	Brain, head, neck, 140	Nifty Net, High ResNet	2-4% 6%, 100%	Siemens biograph 6	SSIM, PSNR, RMSE, SUVmean bias
Weyts et al. (61)	Whole body, 195	Subtle PET 2.5D Unet	45 s, 90 s	VEREOS Philips Health care	SULmax, SULpeak, median IQR, visual eval
Bonardel et al. (3)	Whole body, 100	Subtle PET 2.5D Unet	33%, 50%, 100%	GE discovery MI 4 710, IQ4	CRS, BV, CNR SUVmax, visual eval
Yang et al. (62)	Brain, 15000 images	Cycle GAN supervised, quasi supervised	—	—	RMSE, SSIM, PSNR
Jang et al. (23)	Whole body, 112	Unet, Swin, Restormer	25%, 100%	GE DMI	SSIM, PSNR, CNR
Yu et al. (63)	Whole body, 377=302+15+50	2D, 3D DDPM, 3D UNet	5%, 100%	Siemens Biograph Vision Quadra	SSIM, PSNR
Our	Whole body, 212=160+26+26	SwinIR, ResNet, Unet, 3D MedNeXt, 3D UX-Net, Cycle GAN image prior,	30 s, 60 s, 90 s	GE Discovery 710	RMSE, SSIM, IQR, SUVmax, SUVpeak median bias

An obvious approach for self-supervised PET denoising is to train the model on artificially degraded images (43). CycleGAN is the most popular unsupervised model for PET denoising. The article (28) was the first applied CycleGAN model for whole-body PET denoising. The study (9) also used unsupervised learning. CycleGAN performed better over Unet and Unet GAN in peak signal-to-noise ratio (PSNR) for all human body parts. Sanaat et al. (51) also utilized CycleGAN architecture and demonstrated its performance over ResNet, both trained on 60 studies data set. The ResNet showed, in turn, better results than Unet, which coincides with the results of our experiments. The studies (28, 51) do not reveal the CycleGAN backbone used in their studies; therefore, it remains to be seen if CycleGAN in (28, 51) achieved high performance due to the unsupervised scheme and the adversarial losses or because of difference in the backbone. CycleGAN model applies in different medical image denoising problems such as optical coherence tomography images (39) and low-dose X-ray CT (26, 60). 3D CycleGAN framework with self-attention generates the FC PET image from LC PET with CT aid in the study (29). The study (66) used CycleGAN with Wasserstein loss for stability. Another type of generative models used for PET denoising is diffusion models (17, 45, 46, 57) .

The PET denoising problem is very similar to the PET reconstruction from CT. The study (5) demonstrated that non-contrast CT alone could differentiate regions with different FDG uptake and simulate PET images. To predict three clinical outcomes, using the simulated PET, the article (5) constructed random forest models on the radiomic features. The objective of this experiment was to compare predictive accuracy between the Cycle GAN-simulated and FT PET. ROC AUC for simulated PET achieved to be comparable with ground truth PET—0.59 vs. 0.60, 0.79 vs. 0.82, and 0.62 vs. 0.63. The study (30) denoised CT images by a GAN with the reconstruction loss.

The most popular supervised models [Table 1 in (33)] for PET denoising are ResNet [e.g., (51)] and Unet-style networks [e.g., (53, 55)]. The article (52) used HighResNet, demonstrating that due to PET acquisition's stochastic nature, any LD versions of the PET data would bear complementary/additional information regarding the underlying signal in the standard PET image. This complementary knowledge could improve a deep learning-based denoising framework and [as (52) showed] enhance the quality of FD prediction—PSNR increased from 41.4 to 44.9 due to additional LD images. The study (20) used Swin transformer for FD brain image reconstruction from LC sinograms. The article (23) proposed spatial and channel-wise encoder–decoder transformer—Spatch Transformer that demonstrated better denoising quality over Swin transformer, Restormer, and Unet for 25% low-count PET. An important metric for noise reduction quality is tumor edge preservation. The study (41) showed that CNNs have the same edge preserving quality as bilateral filtering, but it does not provide any quantitative measure of edge preservation.

SubtlePET^TM^ (12) is a commercial product; its official site claims that “SubtlePET is an AI-powered software solution that denoises images conducted in 25% of the original scan duration (e.g., 1 min instead of 4)”. SubtlePET uses multi-slice 2.5D encoder–decoder U-Net (61) optimizing L1 norm and SSIM. The networks were trained with paired low- and high-count PET series from a wide range of patients and from various PET/CT and PET/MR devices (10 General Electric, 5 Siemens, and 2 Philips models). The training data included millions of paired image patches from hundreds of patient scans with multi-slice PET data and data augmentation.

The studies (25, 61, 62) investigated FT 90-s PET reconstruction from LT 30-, 45-, and 60-s images using SubtlePET. The work (61) conducted a study on the efficiency of SubtlePET by comparing denoised LT 45-s PET with FT 90-s PET. The visual analysis revealed a high similarity between FT and reconstructed LT PET. SubtlePET detected 856 lesions for 162 (of 195) patients. Of these, 836 lesions were visualized in both original 90-s PET and denoised 45-s PET, resulting in a lesion concordance rate of 97.7%. The study (3) examined the limits of the SubtlePET denoising algorithm applied to statistically reduced PET raw data from three different last-generation PET scanners compared to the regular acquisition in phantom (spheres) and patient. Enhanced images (PET 33% + SubtlePET) had slightly increased noise compared to PET 100% and could potentially lose information regarding lesion detectability. The PET 100% and PET 50% + SubtlePET were qualitatively comparable regarding the patient data sets. In this case, the SubtlePET algorithm was able to correctly recover the SUVmax values of the lesions and maintain a noise level equivalent to FT images.

The main issue with the studies mentioned above is that it is difficult to compare methods as they use different metrics and data sets. In this study, we have compared various methods, including approaches from other studies and new methods.

**The main contribution of the article are**

- Our comparative experiments of 2D, 2.5D, and 3D models with different backbones and losses trained and tested in the same conditions on a specially collected data set from clinical practice data showed better performance [Root Mean Square Error (RSME) and Structural Similarity Index] of 3D methods.
- We have trained neural networks of the SwinIR (2D and 2.5), 3D UX-Net (3D), and MedNeXt (3D) architectures. Our tests have shown that MedNeXt is the best at restoring an image from 30 s to 90 s and it is approximately equal to 3D UX-Net, and they solve the problem better than all other tested architectures.

## 2 Materials and methods

### 2.1 Whole-body PET data set

We used 212 PET scans from 212 patients obtained during a retrospective study. The data were obtained from scanners calibrated using the NEMA phantom according to the EARL method in June and July 2021. The training subset contains 160 scans with 42,656 images, validation, and test data both consist of 26 scans and 7,126 images, respectively. The patient age is between 21 and 84 years old, and two-thirds of patients are between 49 and 71 years old. The median age is 61; 71% of patients are women. An average height is 1.66 ± STD = 0.09 meters and ranges from 1.47 to 1.94 meters. The weight range is from 34 kg to 150 kg with an average value of 79 ± STD = 18 kg. The body mass index (BMI) is 28.5 ± STD = 6.3.

The number of tumors in the train subset is 521. When excluding 4 patients with more than 50 tumors, the total number of tumors is 139. Of the 160 patients in train subset, 30 do not have any tumors detected. The number of tumors detected in the test set is 74 (for 26 patients, four do not have any tumors), and in the validation set, it is 97 (for another 26, four patients also do not have a tumor). We analyzed the PET scan as a 3D object and, therefore, used all slices to detect the tumor.

There are two ways (5) to simulate low-dose PET—short time frame and decimation. The most common way of decimation is the simulation of a dose reduction by randomized subsampling of PET list-mode data. Another decimation method is randomly sampling the data by a specific factor in each bin of the PET sinogram (50). Short time frames with the corrections taking a shorter amount of time into account will produce images with an SUV uptake similar to the original one. We use short time frame approach in our study collecting PET data with 30, 60 and 90 s/bed positions. All images were collected during the same acquisition session that differs, for example, from studies (50, 51) where the LT images obtained through a separate fast PET acquisition corresponding to the FT scans.

In the data set we used in this study, patients were injected intravenously with 7 MBq/kG [18F]FDG after a 6-h fasting period and blood glucose level testing. The PET data are collected from GE Discovery 710 and were reconstructed using the VPFXS reconstruction method (24). PET frame resolution is 256 × 256. The slice thickness is 3.27 mm, and pixel spacing is 2.73 mm.

The studies are in anonymized DICOM (*.dcm) format, from which one can extract the patient weight, half-life, total dose values and delay Δ*t* between the injection time, and the scan start time for the further SUV calculation.

### 2.2 Methodology

#### 2.2.1 Problem statement

The study aims to assess the quality of the PET denoising for supervised and unsupervised models and find the best model. Unet, ResNet, CycleGAN, pix2pix GAN, SwinIR transformer, MedNeXt, and 3D UX-Net are models to be tested in this research. More details of the network architecture are in the next section.

In academic research, gaussian convolution has been widely recognized as a baseline model for denoising due to its simplicity and proven effectiveness in various denoising tasks. The filter's parameters are crucial for achieving optimal denoising results, and therefore, we optimized them on a validation data set. All models shared the same learning schedule and parameters (with minor differences described in the next section) and, therefore have the same level playing field and could be fairly compared. Table 2 is a systematization of the methods and models used in the study.

**Table 2:** Studied models.

**Type of model**	**GANs unsupervised**	**Supervised**	**GANs + supervised**
Transformer	–	2D&2.5D SwinIR	–
Convolutional transformer-inspired	–	3D MedNeXt, 3D UX-Net	–
Convolutional (Unet, ResNet)	2D CycleGAN	2D&2.5D Unet, ResNet + decoder	2D pix2pix GAN, CycleGAN superv

#### 2.2.2 Denoising quality assessment

Two metric types describe the quality of denoising: a similarity of 2D PET images and concordance of the tumor's SUV characteristics. The metrics for similarity are SSIM (48) and RMSE:


(1) $$\begin{array}{l} \begin{array}{c} \text{RMSE}=\sqrt{\frac{1}{n}\overset{n}{\underset{i=1}{\sum }}{\left(FT_{i}-\hat{LT_{i}}\right)}^{2}} \end{array} \end{array}$$

where *FT*_*i*_ is the i-th measurement, $\hat{LT_{i}}$ is its corresponding prediction, and n is a number of pixels. The SSIM parameters in our study are the same as in scikit-image library. In this report, we used ISSIM=1-SSIM instead of SSIM as ISSIM is more convenient for similar images, and SSIM is higher than 0.9 for most original and denoised PET. We defined relative metrics in the same way as in (50):


(2) $$\begin{array}{l} \text{relRMSE}=1-\frac{\text{RMSE}\left(denoising\left(LT\right),FT\right)}{\text{RMSE}\left(LT,FT\right)} \end{array}$$

the Equation 2 demonstrates the improvement of the denoising method for noised LT image by showcasing the decrease in the discrepancy between the denoised and original image. This equation provides a clear and quantifiable measure of the improvement achieved through the denoising process. The relative ISSIM is defined similarly as Equation 2. The relative metric changes in range from −∞ to 100%. The negative value means that the method has deteriorated the quality of the image, 0%—there are no changes, 100%—the image has been fully denoised and coincides with the original one.

#### 2.2.3 SUV error estimation

**Figure 2:**
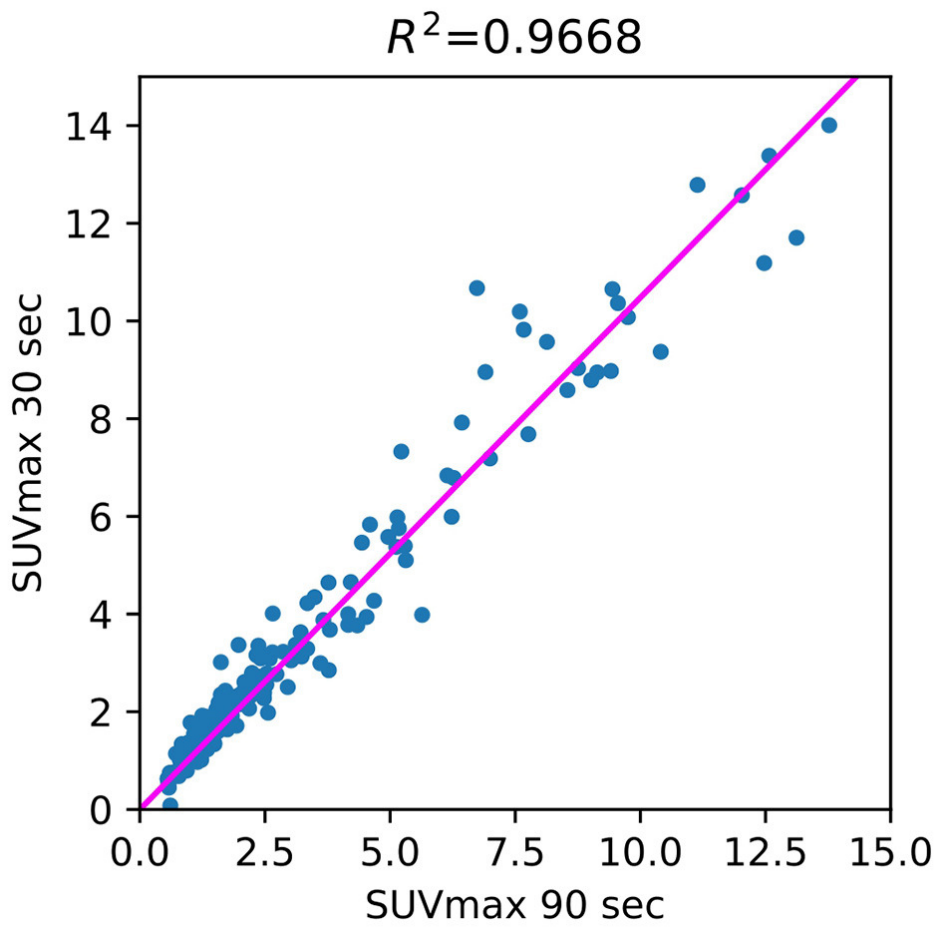
Correlation between 90- and 30-s PET SUVmax (regions of interests).

The use of standard uptake value (SUV) is now commonplace (14) in clinical FDG-PET/CT oncology imaging and has a specific role in assessing patient response to cancer therapy. SUVmean, SUVpeak (58), and SUVmax are the values commonly used in PET studies. There are many ways to estimate the correlation between pairs of SUV values for the FT original PET and denoised PET reconstructed from LT. The most common are bias and STD (Figure 6) in terms of Bland–Altman plots (25, 29, 61) and R2 (Figure 2).

CT is available along with the ground truth PET and could enhance the quality of the tumor segmentation. Instead of employing a radiologist for the malignant tumor detection, we segmented tumors automatically in 3D with the help of nnUnet (21). The pretrained weights are the same as in the AutoPET competition baseline (15). The nnUnet neural network manipulated two channels (PET & CT) input with 400 × 400 resolution. Figure 1 demonstrates tumor segmentation and SUVmax estimation for the region of interest. The CT and PET images are to be resized as they have 512 × 512 and 256 × 256 resolution.

**Figure 3:**
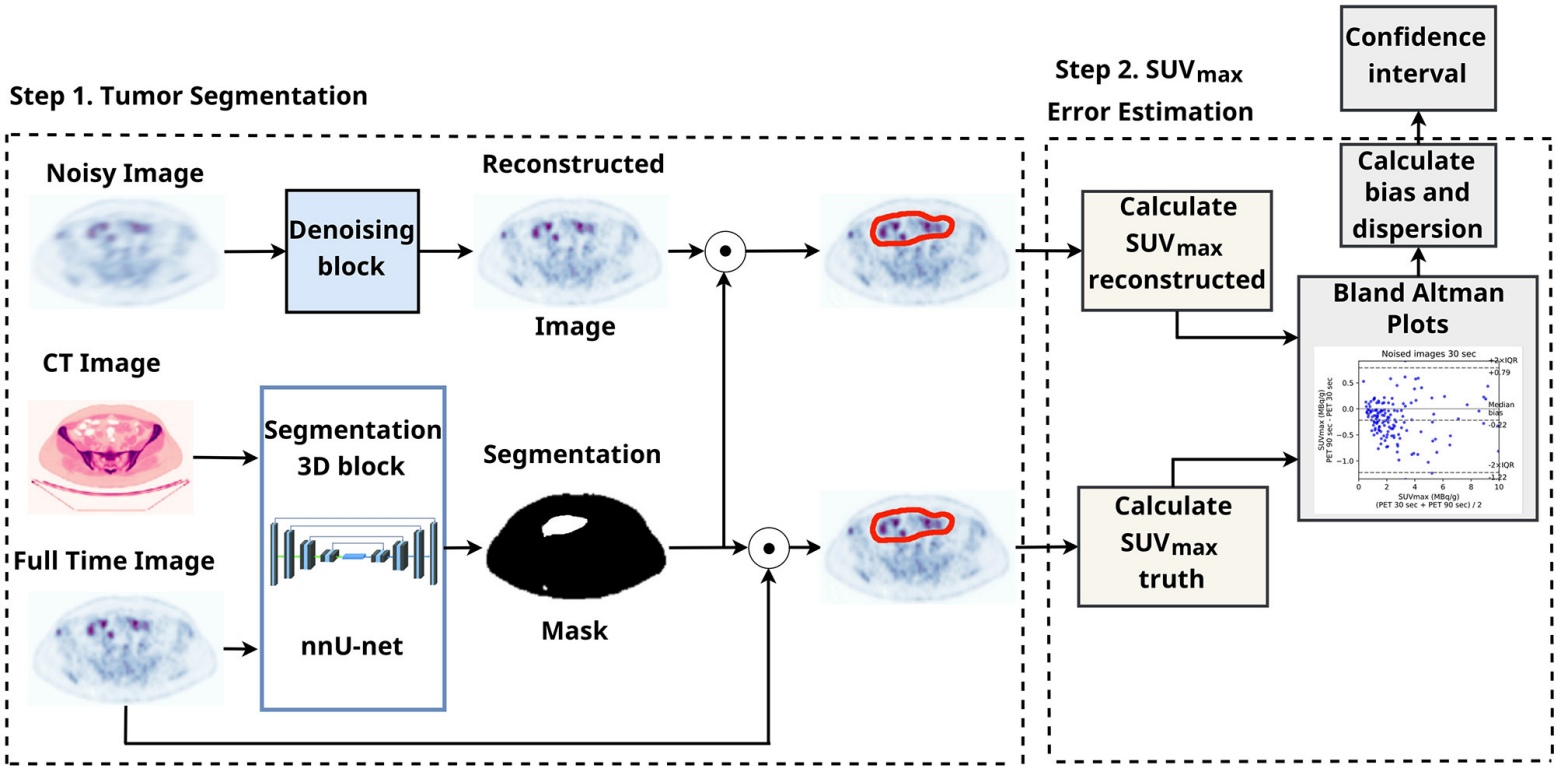
SUV confidence interval estimation scheme with automatic tumor segmentation.

Figure 3 illustrates the SUV confidence interval estimation scheme. After the nnUnet segmentation, cc3d library^1^ extracts 3D connected components and separates different tumors. We excluded from the study tumors with a maximum length of less than 7 mm and an average SUV of less than 0.5. Bland–Altman plot is a standard instrument of data similarity evaluation in biomedical research. The plot operates with the region of interest SUV for original and denoised PET. The Bland–Altman plot's bias and dispersion are indicators of denoising quality and are used in the latest step of the scheme in Figure 3 for the confidence interval assessment. The total number of tumors in validation and test data is 171.

### 2.3 Neural network implementation and training details

#### 2.3.1 2D convolutional networks

Unet and ResNet models, pix2pix, and CycleGAN are based on the pytorch implementation of CycleGAN.^2^ The model parameter numbers are in Table 3. The number of channels in the bottleneck for both models is 64. The Unet model has 54.4 million parameters, the ResNet served as encoder has 11.4 mil. parameters itself, and the decoder has 0.37 mil parameters—11.8 mil parameters in total. The decoder exploits transposed convolutions and does not have skip connections.

**Table 3:** Model parameters.

**Method**	**Parameters m**	**Epoch number**	**Time**	**Max Lr ( × 10^-3)**	**Lr schedule**	**Batch size**	**GPU model**
SwinIR 1 channel, 30 s	11.5	91	1.5d	0.071	Reduce on Plateau	32	NVidia Tesla A100
SwinIR 3 channels, 30 s	11.5	187	2.1d	0.009	Reduce on Plateau	32	NVidia Tesla A100
SwinIR 5 channels, 30 s	11.5	129	1.5d	0.018	Reduce on Plateau	32	NVidia Tesla A100
SwinIR 1 channel, 60 s	11.5	110	1.8d	0.069	Reduce on Plateau	32	NVidia Tesla A100
SwinIR 3 channels, 60 s	11.5	200	2.3d	0.108	Reduce on Plateau	32	NVidia Tesla A100
SwinIR 5 channels, 60 s	11.5	180	2.9d	0.062	Reduce on Plateau	32	NVidia Tesla A100
ResNet 1 channel, 30 s	11.36	89	7.9h	2.178	Reduce on Plateau	32	NVidia Tesla A100
ResNet 3 channels, 30 s	11.4	81	16.0h	1.814	Reduce on Plateau	32	NVidia Tesla V100
ResNet 5 channels, 30 s	11.4	93	19.2h	1.272	Reduce on Plateau	32	NVidia Tesla V100
ResNet 1 channel, 60 s	11.36	45	3.8h	3.314	Reduce on Plateau	32	NVidia Tesla A100
ResNet 3 channels, 60 s	11.4	53	10.9h	2.922	Reduce on Plateau	32	NVidia Tesla V100
ResNet 5 channels, 60 s	11.4	81	16.5h	0.311	Reduce on Plateau	32	NVidia Tesla V100
UNet 1 channel, 30 s	54.4	83	2.4h	1.978	Reduce on Plateau	32	NVidia Tesla A100
UNet 3 channels, 30 s	54.4	72	4.5h	0.927	Reduce on Plateau	32	NVidia Tesla V100
UNet 5 channels, 30 s	54.4	54	3.5h	0.525	Reduce on Plateau	32	NVidia Tesla V100
UNet 1 channel, 60 s	54.4	83	2.4h	2.540	Reduce on Plateau	32	NVidia Tesla A100
UNet 3 channels, 60 s	54.4	63	3.9h	10.664	Reduce on Plateau	32	NVidia Tesla V100
UNet 5 channels, 60 s	54.4	132	8.7h	0.381	Reduce on Plateau	32	NVidia Tesla V100
3D UX-Net, 30 s	53.0	1.3d	9	0.120	Reduce on Plateau	4	NVidia Tesla A100
3D UX-Net, 60 s	53.0	76	9.8d	0.001	Reduce on Plateau	4	NVidia Tesla A100
MedNeXt small, 30 s	5.6	54	6.5d	0.224	Reduce on Plateau	4	NVidia Tesla A100
MedNeXt small, 60 s	5.6	92	11.0d	0.246	Reduce on Plateau	4	NVidia Tesla A100
Pix2pix GAN ResNet	28.25	35	9h	0.2	Cos	32	NVidia Tesla V100
CycleGAN ResNet	114.3	50	46.5h	0.1	Linear	16	NVidia Tesla V100

SwinIR (31) integrates the advantages of both CNN and transformer. On the one hand, CNN has the advantage of processing images of a large size due to the local attention mechanism. On the other hand, it has the benefit of the transformer to model long-range dependency with the shifted window (34). SwinIR exceeded state-of-the-art CNN performance in denoising and JPEG compression artifact reduction. We implemented code from the official SwinIR repository.^3^

SwinIR consists of three modules, namely, shallow feature extraction, deep feature extraction, and high-quality image reconstruction modules. The shallow feature extraction module uses a convolution layer to extract shallow features directly transmitted to the reconstruction module to preserve low-frequency information.

The deep feature extraction module mainly comprises residual Swin Transformer blocks, each utilizing several Swin Transformer layers for local attention and cross-window interaction. In addition, Liang et al. (31) added a convolution layer at the end of the block for feature enhancement and used a residual connection. In the end, shallow and deep features are fused in the reconstruction module for high-quality image reconstruction. The patch size of SwinIR in our training is 32; the window size is 8.

#### 2.3.2 GANs

Pix2Pix and CycleGAN models use PatchGAN (22) with 2.8 million parameters. ResNet, Unet, and CycleGAN models predict the difference between noised and denoised images. The SwinIR model has this difference built into its architecture, like in (38). The Pix2Pix GAN discriminator also used image difference to distinguish between noised and denoised PET. This simple approach applied for the PET denoising improved the results significantly but was used before only in the transformer-based model for CT denoising.

L1 loss is used in all models (except for unsupervised CycleGAN and SwinIR) to optimize the similarity between denoised LT and FT images. SwinIR uses Charbonnier loss (6). Pix2pix GAN also uses Euclidean adversarial loss. In the original CycleGAN paper (67), identity mapping loss


(3) $$\begin{array}{l} ident=\overset{n}{\underset{i=0}{\sum }}||denoised\left(FT_{i}\right)-FT_{i}{||}_{L1}+||noised\left(LT_{i}\right)-LT_{i}{||}_{L1} \end{array}$$

helps preserve the color of the input painting. The loss prevents the network from denoise the FT image and vice versa. Park et al. (47) claims that more weights for the cycle consistency loss and identity loss made the CycleGAN model translate the blood-pool image close to the actual bone image. We will investigate the influence of identity loss on PET denoising.

CycleGAN is an unsupervised method. Therefore, its usage is beneficial if there is a lot of unpaired data in both domains. However, getting paired data with different PET acquisition times is an ordinary task that could be done automatically without any additional action on a patient. The study (62) showed that the use of the additional supervised reconstruction loss (4) in CycleGAN makes the training stable and considerably improves PSNR and SSIM


(4) $$\begin{array}{l} rec\text{\_}loss=\frac{1}{n}\overset{n}{\underset{i=0}{\sum }}||denoised\left(LT_{i}\right)-FT_{i}{||}_{L1}. \end{array}$$

We used supervised CycleGAN as an upper boundary for ISSIM and RMSE metrics that unsupervised CycleGAN could achieve with the image prior loss. We also studied its effect on SUVmax error. We trained CycleGAN with identity. Zhu et al. (67) and image prior (60) losses in addition to adversarial and cycle consistency losses. Image prior loss


(5) $$\begin{array}{l} img\text{\_}prior=\overset{n}{\underset{i=0}{\sum }}||denoised\left(LT_{i}\right)-LT_{i}{||}_{L1}. \end{array}$$

is based on the assumption of similarity between LT noised and FT original PET slices. It performs a regularization over CycleGAN generators, preventing them from generating denoised PET images that are very different from the original one.

#### 2.3.3 2.5D and 3D methods

The 2.5D models have a similar architecture as their 2D counterparts. The difference is in the number of input channels (2, 44). To predict the i^th^ slice denoising, we used *k* adjacent slices—2*k*+1 slices in total. These slices were fed into our models as 2*k*+1 channel images. The increase in input channel number slightly raised GPU memory and time consumption. We trained the 2.5D model in the same manner as we trained the 2D model. The only difference is that we used only the central channel of SwinIR output as the result.

We employed novel 3D ConvNeXt-like architectures, namely, MedNeXt (49) small (5.6 million parameters), and 3D UX-Net (27). These models draw inspiration from the transformer architecture, but it is a pure convolutional model. The authors of MedNeXt adopted the general design of ConvNeXt block for 3D-UNet-like architecture. The MedNext architercture utilizes depthwise convolution, GroupNorm, and Expansion and Compression layers. The novelty of the model is the usage of residiual inverted bottlenecks in place of regular up and downsampling blocks as well as the technique of iteratively increasing kernel size. We fed a 32 PET slices chunk into the input of the network.

#### 2.3.4 Training details

The training parameters are in Table 3. The hyperparameter tuning is done on the validation data set by maximizing SSIM with optuna library. SSIM is preferrable over L1 and RMSE as it, more than other metrics, coincides with human perception and makes denoised PET look similar to the original (48).

We considered identity and image prior loss coefficients between 0 and 30 and weight decay for Unet in the 0.001–0.2. The ISSIM dependence of image prior loss coefficient looks the same as in (60). The quality of denoising is stable to the identity loss coefficient but could deteriorate up to 20% of its value when choosing a coefficient higher than 18.

The augmentations used in training are horizontal and vertical flips. The augmentations did not significantly improve metrics for ResNet, but they made the training process more stable. In contrast, CycleGAN with ResNet backbone metrics slightly dropped when trained with augmented images. The Unet performance improved significantly after applying augmentations but still lagged behind ResNet; this could be partly due to overfitting as Unet has more parameters than ResNet.

Adam is an optimizer for the training process. Unet was trained with weight decay = 0.002 to prevent overfitting improving relative ISSIM from 27.8% to 29.0%. The usage of dropout has a similar effect. The learning rate was chosen individually to achieve the best performance for each model. We trained supervised methods and pix2pix GANs with ResNet backbones using a cos learning rate schedule, max lr = 0.0002 for 35 epochs. CycleGAN training includes 30 epochs with a constant learning rate of 0.0001, which is linearly reduced to zero for the following 15 epochs. The Optuna library helped to fit the optimal learning rate schedule for SwinIR.

We trained models with batch size 32 except CycleGAN.The original CycleGAN (67) used batch size 1. Unlike the original work in the recent study (37), the batch size that generates the best PSNR value is 64, using the initial learning rate. The experiments demonstrated that the batch size does not have to be 1 or 4, but it depends on the data set size and the problem type. Therefore, CycleGAN was trained with batch size 16.

## 3 Results

**Figure 4:**
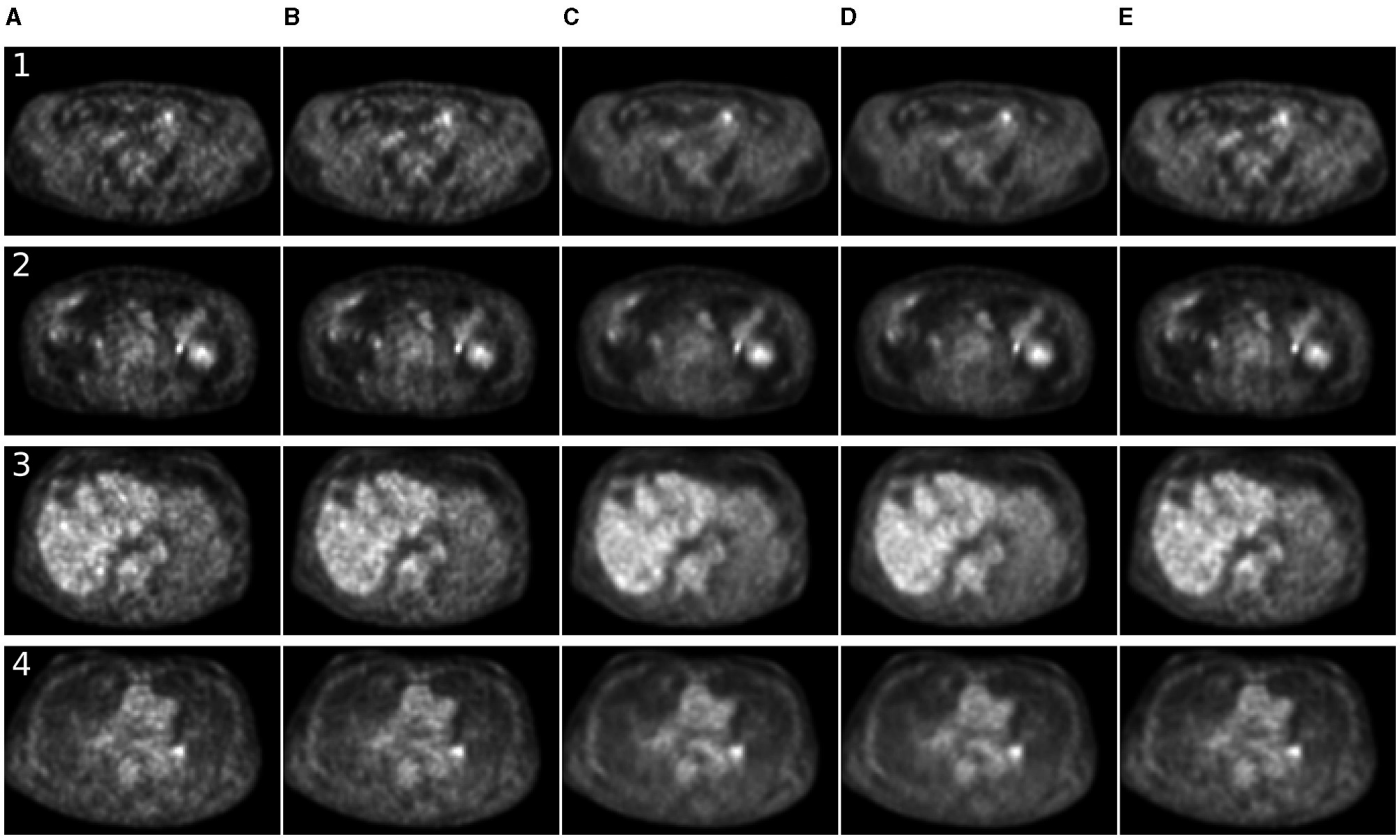
Comparison of denoising methods applied on low-time (30 s) PET reconstruction. **(A)** Low-time PET (30 s). **(B)** Full-time PET (90 s). **(C)** Reconstructed PET (by SwinIR 5 channels). **(D)** Reconstructed PET (by MedNeXt small). **(E)** Reconstructed PET (by Gaussian filter).

**Figure 5:**
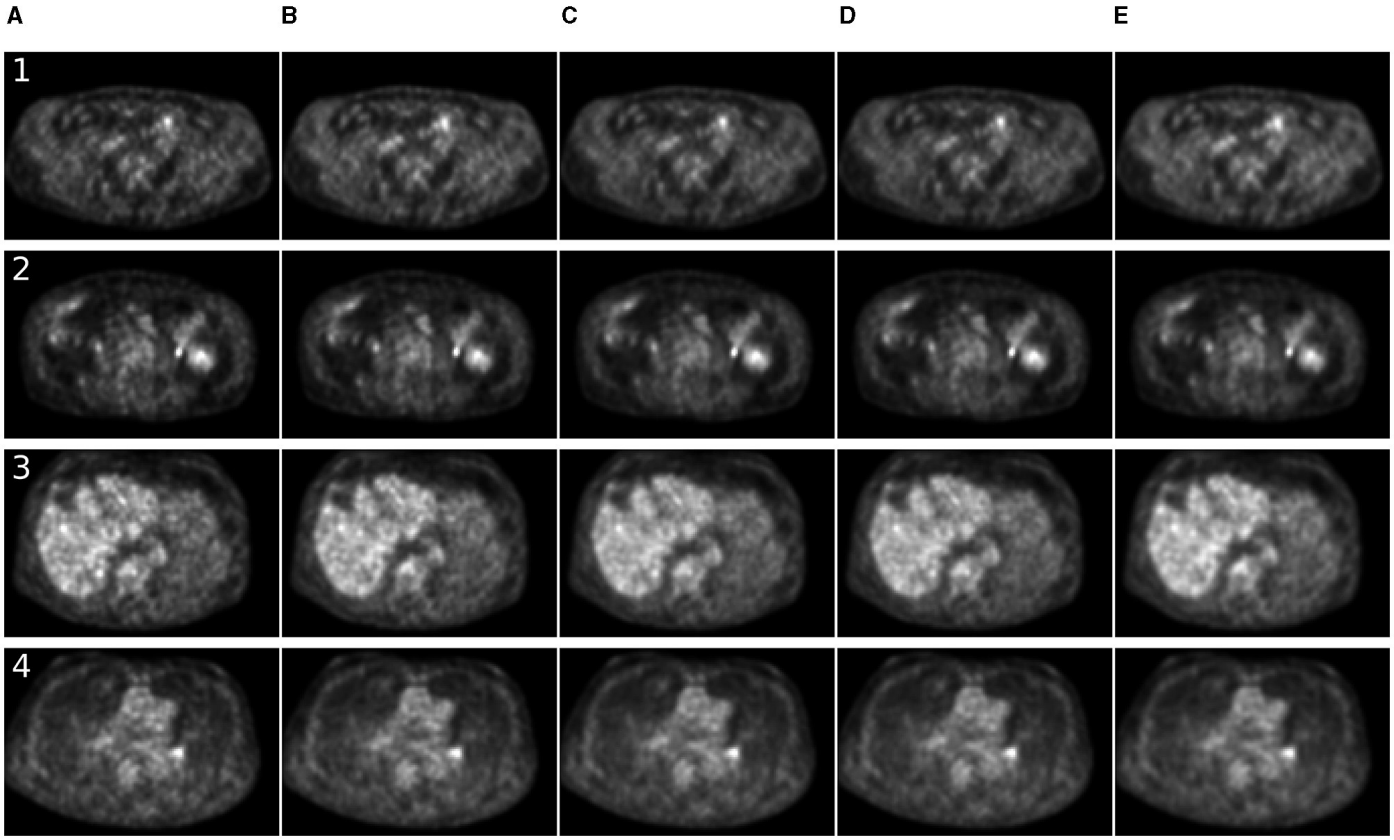
Comparison of denoising methods applied on low-time (60 s) PET reconstruction. **(A)** Low-time PET (60 s). **(B)** Full-time PET (90 s). **(C)** Reconstructed PET (by SwinIR 5 channels). **(D)** Reconstructed PET (by MedNeXt small). **(E)** Reconstructed PET (by Gaussian filter).

For 7 MBq/kg injected activity and 90-s FT, the 3D methods have shown exceptional results in enhancing SSIM and MSE metrics, surpassing 2D methods. The 2.5D approach showed superiority over 2D counterparts for SwinIR only. For 60-s PET, SwinIR 2.5D has slightly surpassed 3D methods. When evaluating the SUV error, 3D methods demonstrated comparable results to the 2D methods. Unlike in the study (65), adding CT information as a second channel, along with PET, did not improve the quality of denoising process. The examples of denoised images are in Figures 4, 5.

**Figure 6:**
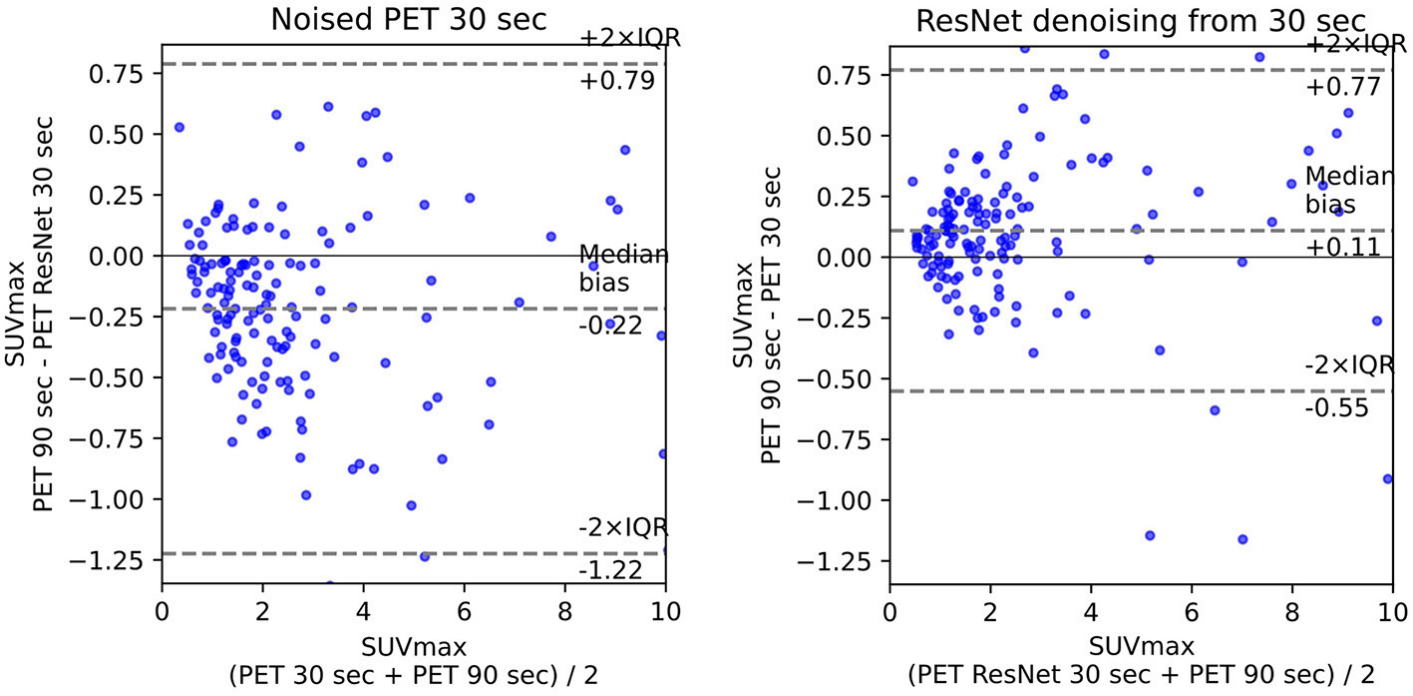
Bland–Altman plot for 30 s PET before and after denoising.

The ISSIM and RMSE metrics for 30 sec and 60 sec PET are in Tables 4, 5, respectively. If the value in a Table has ±, it means that the network we trained independently five times to estimate the confidence intervals (95% confidence level). Tables 6, 7 represent discrepancy [Median bias and Interquartile Range (IQR) values for the Bland–Altman plot, Figure 6] in SUVpeak and SUVmax estimation. We preferred median and IQR over mean and STD like (61) did as these metrics are more robust to the outliers.

**Table 4:** Similarity of 2D PET images—original 90 s and reconstructed from 30 s.

**Model**	**Absolute values**	**Relative values**	
	**(1 - SSIM) (**×**10^-2)**	**RMSE**	**1 - SSIM**
30 vs. 90	8.58	0%	0%
Gauss conv	7.17	12.6%	15.3%
SwinIR 1 channel	6.06_±0.04_	22.9_±0.28_%	29.7_±0.50_%
ResNet 1 channel	5.77_±0.06_	24.4_±0.59_%	32.9_±0.82_%
UNet 1 channel	6.09_±0.04_	22.0_±0.46_%	29.1_±0.32_%
Cycle GAN sup	6.14_±0.12_	20.1_±1.0_%	28.1_±1.0_%
CycleGAN	6.29_±0.12_	19.2_±1.2_%	26.6_±1.3_%
CycleGAN identity	6.22_±0.17_	20.4_±1.5_%	27.3_±2.2_%
CycleGAN image prior	6.24_±0.07_	19.1_±0.6_%	27.1_±0.78_%
SwinIR 3 channels	6.07	23.99%	29.4%
SwinIR 5 channels	5.56	27.1%	35.9%
ResNet 3 channels	5.92	23.5%	31.3%
ResNet 5 channels	5.84	24.03%	32.3%
UNet 3 channel	5.96	22.7%	30.4%
UNet 5 channel	5.94	22.6%	30.7%
MedNeXt small	**5.36**	28.07%	**38.2%**
3D UX-Net	5.37	**28.15%**	38.0%

Bold values are the best values.

**Table 5:** Similarity of 2D PET images—original 90 s and reconstructed from 60 s.

**Model**	**Absolute values**	**Relative values**	
	**(1 - SSIM) (**×**10^-2)**	**RMSE**	**1-SSIM**
60 vs. 90	2.61	0%	0%
Gauss conv	2.44	3.21%	5.3%
SwinIR 1 channel	2.27_±0.011_	9.0_±0.27_%	12.7_±0.5_%
ResNet 1 channel	2.24_±0.016_	**9.3**_±0.42_%	13.8_±0.65_%
UNet 1 channel	2.29_±0.015_	8.4_±0.28_%	12.4_±0.53_%
SwinIR 3 channels	2.20	11.2%	16.2%
SwinIR 5 channels	**2.172**	**11.7%**	**17.1%**
ResNet 3 channels	2.28	8.6%	12.6%
ResNet 5 channels	2.27	9.1%	13.2%
UNet 3 channels	2.29	8.47%	11.96%
UNet 5 channels	2.27	8.74%	12.9%
CycleGAN	2.33_±0.011_	6.6_±0.27_%	10.3_±0.23_%
Cycle GAN sup	2.32_±0.018_	6.8_±0.44_%	10.8_±0.84_ %
CycleGAN identity	2.32_±0.011_	7.1_±0.54_%	11.0_±0.27_%
CycleGAN image prior	2.33_±0.013_	6.8_±0.51_%	10.7_±0.49_%
MedNeXt small	2.174	11.4%	**16.9**%
3D UX-Net	2.22	10.2%	14.6%

ResNet backbone. Bold values are the best values.

**Table 6:** SUVpeak and SUVmax characteristics original.

**Model**	**SUVpeak**			**SUVmax**		
**Method**	**(1 - R**^2^) **(**×**10**^−3^**)**	**Median bias (**×**10**^−2^**)**	**IQR (**×**10**^−2^**)**	**(1 - R**^2^) **(**×**10**^−2^**)**	**Median bias (**×**10**^−2^**)**	**IQR (**×**10**^−1^**)**
30 vs. 90	8.48	1.52	12.62	3.061	-21.78	5.031
Gauss convol	9.53	**0.85**	11.12	8.282	9.71	4.667
SwinIR 1 channel	7.8	2.6	**9.07**	**1.8**	16.2	3.5
ResNet 1 channel	8.8_±1.9_	1.7_±1.1_	10.7_±0.6_	2.1_±0.5_	11.0_±12.0_	3.5_±0.9_
Unet 1 channel	9.0_±1.6_	2.0_±2.4_	10.3_±0.9_	19.6_±4.4_	13.1_±1.9_	3.8_±0.7_
SwinIR 5 channels	10.93	2.65	10.37	2.933	19.92	3.845
Cycle GAN sup	8.0_±0.5_	1.8_±0.5_	10.6_±0.3_	2.1_±0.21_	6.2_±4.0_	**3.4**_±0.28_
CycleGAN	7.6_±0.25_	1.3_±1.2_	10.9_±0.6_	2.1_±0.37_	2.4_±1.6_	3.8_±0.5_
CycleGAN identity	**7.5**_±0.23_	0.9_±0.8_	10.7_±0.9_	1.96_±0.12_	5.4_±4.9_	3.6_±0.4_
CycleGAN image prior	8.0_±0.25_	2.1_±0.7_	11.3_±0.3_	2.12_±0.27_	**1.4**_±2.9_	3.9_±0.6_
MedNeXt small	9.51	2.55	9.83	2.22	19.97	3.5

PET reconstructed from 30 s with respect to the original 90-s PET. Bold values are the best values.

**Table 7:** SUVpeak and SUVmax characteristics original.

**Model**	**SUVpeak**			**SUVmax**		
**Method**	**(1 - R**^2^) **(**×**10**^−3^**)**	**Median bias (**×**10**^−3^**)**	**IQR (**×**10**^−2^**)**	**(1 - R** ^2^) **(**×**10**^−3^**)**	**Median bias (**×**10**^−2^**)**	**IQR (**×**10**^−1^**)**
60 vs. 90	2.19	-0.2	5.92	5.93	-5.95	2.489
Gauss convol	2.2	1.3	6.46	16.4	3.01	2.545
SwinIR 1 channel	2.4_±0.11_	6.6_±2.5_	5.6_±0.27_	**3.8**_±0.47_	6.6_±1.3_	2.47_±0.48_
UNet 1 channel	**2.0**_±0.15_	3.0_±9.0_	**5.4**_±0.7_	5.4_±0.4_	2.9_±1.5_	2.41_±0.32_
ResNet 1 channel	2.1_±0.021_	5.36_±4.9_	5.6_±0.6_	5.3_±0.25_	2.9_±2.7_	**2.16**_±0.29_
SwinIR 5 channels	2.28	10.1	5.65	4.36	8.62	2.317
Cycle GAN sup	2.1_±0.05_	**−0.06**_±3.4_	5.6_±0.4_	5.5_±0.3_	-1.3_±0.9_	2.41_±0.08_
CycleGAN	2.07_±0.04_	0.3_±2.3_	5.4_±0.4_	5.7_±0.4_	-1.0_±1.2_	2.49_±0.21_
CycleGAN identity	2.07_±0.018_	-2.8_±2.1_	5.46_±0.18_	5.6_±0.4_	**−0.3**_±1.0_	2.45_±0.09_
CycleGAN image prior	2.09_±0.018_	-1.2_±3.3_	5.81_±0.24_	5.3_±0.13_	-0.7_±1.1_	2.42_±0.17_
MedNeXt small	2.5	4.9	5.7	4.5	8.29	2.5

PET reconstructed from 60 s with respect to the original 90 s PET. ResNet backbone. Median values. Bold values are the best values.

The prediction of the difference between noised and denoised images rather than the prediction of the denoised image itself significantly (twice as much for ISSIM metric) improved the quality of denoising as it is easy for the network to produce noise rather than images (7).

### 3.1 Comparison of 2D and 2.5D methods

ResNet has the best image similarity metrics among 2D methods for both weakly noised (60 s) and strongly noised (30 s) PET. SwinIR and Unet follow it. The quality of SwinIR PET restoration is better than that of Unet. At the same time, Jang et al. (23) demonstrates better Swin performance over Unet on 25% low-count data. This fact indicates that convolutional layers of SwinIR for shallow feature extraction improve reconstruction quality compared to the pure Swin transformer architecture.

The 2.5D approach did not improve the denoising quality for ResNet but significantly improved it for SwinIR with five layers (±2 slices around the slice of interest). This could have happened due to differences in Transformer and convolution architectures. Transformers are better at mixing information between channels. SwinIR with five layers have a similar performance as 3D MedNext for 60-s PET in SSIM and MSE but lagged significantly behind in SUVmax estimation.

### 3.2 Comparison of GAN performance

ResNet backbone with convolutional decoder without skip connection outperformed Unet and SwinIR in all cases, that is why in Table 4 and the following tables, we presented the results for CycleGAN and Pix2pix GAN for the ResNet backbone only. Pix2Pix GAN (ResNet + PatchGAN discriminator) without distance loss produces realistic denoised images with low metrics. The SSIM steadily improves while the distance loss coefficient increases and reaches its plateau when the GAN degrades to a simple supervised model, as distance loss outweighs adversarial losses. Our Pix2Pix GAN did not show a higher quality over other methods as 3D CVT-GAN in (64), or BiC-GAN (13) for the brain's PET synthesis.

As the original Pix2Pix paper (22) mentioned, the random input *z* does not change the GAN results, and the model is indeed deterministic. We concluded that the Pix2Pix GAN model is inappropriate for the denoising problem as adversarial loss improves image appearance instead of SSIM. So, it produces realistic images that are far from the original images. For that reason, we have not included pix2pix GAN metrics in Tables 4–7. We trained PixPix GAN with the coefficient by distance loss of 10.0 as it does not outweigh the adversarial loss.

Unsupervised CycleGAN provides weaker image similarity metrics than supervised. The optimal coefficient for the identity loss is 2.2, and the image prior loss is 9.2. For the distance loss in supervised CycleGAN, we used the same coefficient as for image prior loss was 9.2. For the reduced 30% data set, the impact of the image prior loss is the same as in (60). However for the full-size data set, the image prior loss effect is less pronounced. The reason is that for the small-size data set, the image prior loss works as regularization and prevents overfitting. For example, the size of the data set in (60) is only 906 CT images with 512 × 512 resolution.

The image prior loss improves the quality and stability of the reconstruction. The identity loss has a more profound effect on the SSIM and RMSE metrics than the image prior loss for the weakly noised PET (Table 5). We used the same coefficient for image prior loss as for the distance loss in supervised CycleGAN - 9.2. Supervised CycleGAN image similarity metrics lie between supervised and unsupervised methods but have the advantage of CycleGAN estimating SUVmax with lower bias and dispersion. Our results contradict (28) where CycleGAN outperformed supervised Unet and Unet GAN. That could stem from the small size of the data set used in (28), or the reason is the usage in CycleGAN backbone other than Unet.

## 4 Discussion

The results of experiments showed 3D MedNext is the best model for PET denoising, though 2.5D SwinIR slightly outperforms it in MSE for 60-s PET. The supervised methods produce reconstructed PET with positive SUVmax bias (Figure 6) because they flatten the signals too much. On the other hand, CycleGAN family methods have predominantly negative or around zero bias such as SubtlePET algorithm (61), which is an advantage of unsupervised methods.

The SSIM metric was an optimization goal for the network, so the model having the lowest ISSIM and RMSE results does not necessarily produce the best SUV reconstruction for tumors. In (52), the SUVmean bias was not improved by HighResNet (LD contains only 6% of the FD) even though the PSNR and SSIM of the reconstructed image were better than the LD. There is also a high variation in SUVs for the same model trained several times.

Due to biological or technological factors, SUV may significantly differ from one measurement to another (1, 11, 32). For example, technological factors include inter-scanner variability, image reconstruction, processing parameter changes, and calibration error between a scanner and a dose calibrator. An example of biological factors are respiratory motion (18) like cardiac motion, and body motion. The LT denoising is acceptable if its error on SUV values is smaller than SUV variations from different FT measurements.

We did use SUVmax and SUVpeak measurements as these are metrics commonly used by clinicians for follow-up FDG-PET/CT scans and therapy response evaluation (40). The studies (4, 35, 56) reported high ΔSUVmax between two FDG acquisitions. One should consider the details of these experiments to compare them with the results of our study. The uncertainty estimation of the PET reconstruction (10) is also an important topic for the future research.

The recent work (11) conducted the following experiment. The six phantom spheres of 10–37 mm diameters were filled with the concentration 20.04 MBq/ml. The FT 150-s mode was divided into subsets of shorter frames varying from 4 to 30 s. The SUVmax monotonically increases with sphere's diameter. The ratio of the standard SUVmax deviation to its average value for 30-s PET is approximately 15% for a 10 mm sphere and the confidence interval length achieves up to 0.5 kBq/ml. The experiment (11) does not take into account biological and most of technical factors (1), so the final SUVmax discrepancy between two PET could achieve higher values. The aforementioned estimation of SUVmax discrepancy for the same tumor between two PET acquisitions shows that SUVmax denoising error for the 30- and 60-s PET achieved in our study lies in the acceptable range.

It is a matter of discussion on which metric—SUVmax error or visual similarity should be given priority. Image similarity metrics provide visual information that can help doctors determine whether a tumor is malignant or benign. Image comparison also helps clinicians assess treatment efficacy by comparing pre-treatment images with post-treatment ones to measure any changes due to therapy intervention. On the other hand, SUVmax error provides quantitative data regarding how much a tumor has reduced in size after treatment interventions have been applied; this allows physicians an objective way of evaluating treatments' effectiveness without relying solely on subjective visual assessments from image comparisons alone.

## 5 Conclusion

PET denoising may allow for reducing an injected dose or increasing the scanner's throughput. We reconstructed PET with a reduced acquisition time of 30 and 60 s and compared it with the original full-time 90-s PET for 7MBq/kg injected activity. The AI models reduced PET denoising for 38% (100%–restoration to original image) in the SSIM metric for 30-s PET and for 17% for 60-s PET. The SUVmax discrepancy for the 30- and 60-s PET achieved in our study lies in the acceptable range.

We trained and tested MedNeXt, 3D UX-Net, SwinIR, Unet, ResNet, and CycleGAN with ResNet backbone and different auxiliary losses. The 3D MedNeXt approach has shown the best results in enhancing SSIM and MSE metrics. The supervised denoising methods have significantly better RMSE and ISSIM than unsupervised ones. This result differs from previous studies claiming that CycleGAN surpasses Unet and ResNet. The ResNet reconstructs PET images with the lowest RMSE and ISSIM, outperforming 2D SwinIR and Unet, but lags significantly behind 2.5 SwinIR with five channels. Supervised CycleGAN achieved the lowest SUVmax error after PET denoising. The SUVmax error of the reconstructed PET is comparable with the reproducibility error due to biological or technological factors. Adding CT information to PET does not improve denoising quality.

## Data Availability

The datasets presented in this article are not readily available because the data that support the findings of this study are available from PET Moscow region, but restrictions apply to the availability of these data, which were used under license for the current study, and so are not publicly available. Data are however available from the authors upon reasonable request. Requests to access the datasets should be directed to Ivan Kruzhilov, iskruzhilov@sberbank.ru.

## Footnotes

^1^ https://github.com/seung-lab/connected-components-3d/

^2^ https://github.com/junyanz/pytorch-CycleGAN-and-pix2pix

^3^ https://github.com/JingyunLiang/SwinIR
